# Mendelian randomization rules out the causal relationship between serum lipids and cholecystitis

**DOI:** 10.1186/s12920-021-01082-y

**Published:** 2021-09-17

**Authors:** Hongqun Yang, Lanlan Chen, Kaiyu Liu, Chengnan Li, Haitao Li, Kezhen Xiong, Zehan Li, Chuang Lu, Wei Chen, Yahui Liu

**Affiliations:** 1 Secondary Department of Hepatobiliary and Pancreatic Surgery, The First Hospital of Jilin University, Changchun, 130021 China; 2 Clinical Medical School, Jilin University, Changchun, 130021 Jilin China

**Keywords:** Mendelian randomization, Cholecystitis, Serum lipids, Cholesterol, Triglycerides

## Abstract

**Background:**

The relationship between serum lipids and cholecystitis is still under investigation. To examine the causal effect of serum lipids on cholecystitis using the Mendelian randomization method.

**Methods:**

We conducted univariable Mendelian randomization (MR) analyses using summary statistics from two independent genome-wide association studies (GWAS) on serum lipids (n = 132,908) and cholecystitis (n = 361,194). Mainly, the inverse-variance weighted (IVW) method was utilized to combine each SNP’s causal estimation, and the MR-Egger was adopted as a complementary method, together with the weighted median. Cochrane’s Q value was employed to appraise heterogeneity. The MR-Egger intercept and MR-PRESSO were used to detect the horizontal pleiotropy.

**Results:**

Our univariable results displayed a minor protective effect of serum low-density lipoprotein (LDL) cholesterol (OR [95% CI] = 0.9984483 [0.9984499, 0.9984468]; *p* = 0.008) on cholecystitis. No significant causal effect of total cholesterol (TC) (OR [95% CI] = 0.9994228 [0.9994222, 0.9994233]; *p* = 0.296), triglycerides (OR [95% CI] = 0.9990893 [0.9990882, 0.9990903]; *p* = 0.238) and high-density lipoprotein (HDL) cholesterol (OR [95% CI] = 0.9997020 [0.9997017, 0.9997023]; *p* = 0.565) was found on cholecystitis.

**Conclusion:**

These findings suggest that LDL cholesterolhas a slight protective effect on cholecystitis, which can be easily affected by confounding factors. TC, triglycerides and HDL cholesterol don’t have causal effect on cholecystitis. The protective effect of serum lipids on cholecystitis, though possible, remain less certain.

**Supplementary Information:**

The online version contains supplementary material available at 10.1186/s12920-021-01082-y.

## Introduction

Cholecystitis is one of the most common diseases of the gallbladder. According to a comprehensive survey, 262,411 hospitalizations occurred in 2000 for cholecystitis [1]. Fortunately, the mortality of cholecystitis is only at 0.6% [1]. Cholecystitis could be roughly classified as acute cholecystitis acalculous cholecystitis and chronic cholecystitis. Cholecystectomy is the gold standard treatment of all kinds of cholecystitis. Although it is not a big surgery, it puts a financial and physical burden on patients. Therefore, the prevention of cholecystitis is of vital importance. Many risk factors were reported by the previous study such as obesity, old age, female gender, long-term total parenteral nutrition, diabetes, etc. [2]. However, whether serum lipids are risk factors for cholecystitis is still under investigation. Furthermore, the current results are controversial. For example, in univariable analyses conducted by Mohr et al. [3], they found a negative association between high-density lipoprotein (HDL) cholesterol, low-density lipoprotein (LDL) cholesterol, and cholecystitis while a case–control study conducted by Wang et al. [4] reported that HDL cholesterol was positively associated with cholecystitis. Thus, we hope to find out the causal relationship between blood lipids and cholecystitis. However, it is difficult to conduct a randomized control study (RCT) to investigate the causal relationship. Therefore, we carried out a Mendelian randomization analysis to overcome the limitations of the previous study.

Mendelian randomization (MR) is a method using genetic variants to determine whether there exists a causal relationship between the exposure (usually a risk factor) and the outcome [5, 6]. The random assortment of genetic variants follows the law of Mendelian. As a result, the allocation of exposure for each individual is achieved randomly, which is the fundament of MR study. Numerous MR studies have been published in recent years where some of them even overturned widely accepted opinions. For example, the MR analysis of Holmes et al. [7] ruled out the causal effect of HDL cholesterol on coronary heart disease. MR analysis is a good way to overcome difficulties of conventional research such as confounders, loss of follow-up, time-consuming. The effect of the instrumental variables (IV) lasts for the whole life even before the birth of the individual. It is exactly suitable for the investigation of the lifetime exposure’s effect on the target disease. Compared to conventional observational studies, MR analysis is more convincing and reliable.

Here, we used single nucleotide polymorphisms (SNP) associated with TC, HDL cholesterol, LDL cholesterol, and triglyceride as instrument variables to conduct MR analysis. We extracted SNPs from the genome-wide association studies (GWAS) from the GLGC consortium to investigate the effect of blood lipids on cholecystitis [8]. The levels of TC, HDL cholesterol, LDL cholesterol, and triglycerides were treated as the exposure separately in the univariable MR analysis to investigate its causal effect on cholecystitis. Thus, we hope to disentangle the complex relationship between blood lipids and cholecystitis.

## Methods

### Data source

Data involved in this study are publicly available. We extracted the exposure data from the Global Lipids Genetics Consortium (GLGC, http://lipidgenetics.org/). This GWAS study contained summary statistics from 45 studies. Among them, 37 studies consisted of individuals of European ancestry (n = 114,230). Another 9 studies consisted primarily of individuals with non-European ancestry: two studies of South Asian descent (n = 1516, n = 3385); two studies of East Asian descent (n = 1771, n = 7044); five studies of recent African ancestry, from Uganda (n = 1687), from the Caribbean (n = 426), and the United States (n = 1614, n = 397, n = 838). The exposure data was given in per SD unit. To identify outlier studies, average standard errors for association statistics from each study were plotted against the study sample size. Allele frequencies were scrutinized to guarantee all analyses using the same strand assignment of alleles. Reported statistics and allelic effects were consistent with published findings for known loci. Genomic control values for study-specific analyses were < 1.20. Variants whose minor alleles were observed < 7 times were excluded. Phenotype distribution, proportion of phenotype variance explained by instruments were provided in Table 1.

**Table 1:** The descriptive statistics of instrument variable and phenotype distribution

Descriptive statistics of instrument variable						Publication for instruments
Phenotype	Mean ± SD	Units	Variance explained	Sample size	Consortium	
Cholesterol	213.28 ± 42.6	SD (mg/dL)	0.16	187,365	GLGC	Willer et al. [8]
HDL-cholesterol	53.3 ± 15.5	SD (mg/dL)	0.17	187,167	GLGC	Willer et al. [8]
LDL-cholesterol	133.6 ± 38.0	SD (mg/dL)	0.15	173,082	GLGC	Willer et al. [8]
Triglycerides	140.85 ± 87.8	SD (mg/dL)	0.13	177,861	GLGC	Willer et al. [8]

The summary statistics associated with cholecystitis were extracted from the UK biobank (www.nealelab.is/uk-biobank). They filtered from 487,409 individuals down to 337,199 individuals. Individuals included in the GWAS analysis of the UK biobank were restricted to European descent. Individuals who met any of the following criteria were excluded: (1) closely related individuals, (2) individuals with sex chromosome aneuploidies, (3) individuals who had withdrawn consent from the UK biobank study. Over 92 million imputed autosomal SNPs were available for the GWAS analysis. SNPs with minor allele frequency > 0.1% and Hardy–Weinberg equilibrium (HWE) *p* value > 1 × 10^−10^ were included. Finally, 10.8 million SNPs remained for analysis.

### IV selection (F statistics)

All the SNPs were tested for association with the trait of interest at a genome-wide significant level (*p* < 5 × 10^−8^). SNPs missing in summary statistics of outcome were removed. For the sake of reducing the missing SNPs we selected proxy SNPs (European populations) in high LD (r^2^ > 0.8). We excluded the weak instrument variable. The weak instrument variable was defined as the F statistic less than 10. The F statistic is usually quoted as a measure of the strength of an instrument variable. SNPs that failed to pass the leave-one-out analysis were eliminated. All the SNPs were scrutinized for linkage disequilibrium (LD) according to the criterion as r^2^ < 0.01, kilobase (kb) > 10,000, clumping threshold < 5 × 10^−8^. Palindromic SNPs were excluded from this research. MR-PRESSO global test, outlier test, and distortion test were conducted to identify and remove SNPs with horizontal pleiotropy. Compared to other tests of pleiotropy, all three tests of MR-PRESSO have higher power to detect horizonal pleiotropy except for the perfectly overlapping samples [9]. After series of strict filtration the rest of SNPs were regarded as qualified IVs.

### Main design

**Fig. 1:**
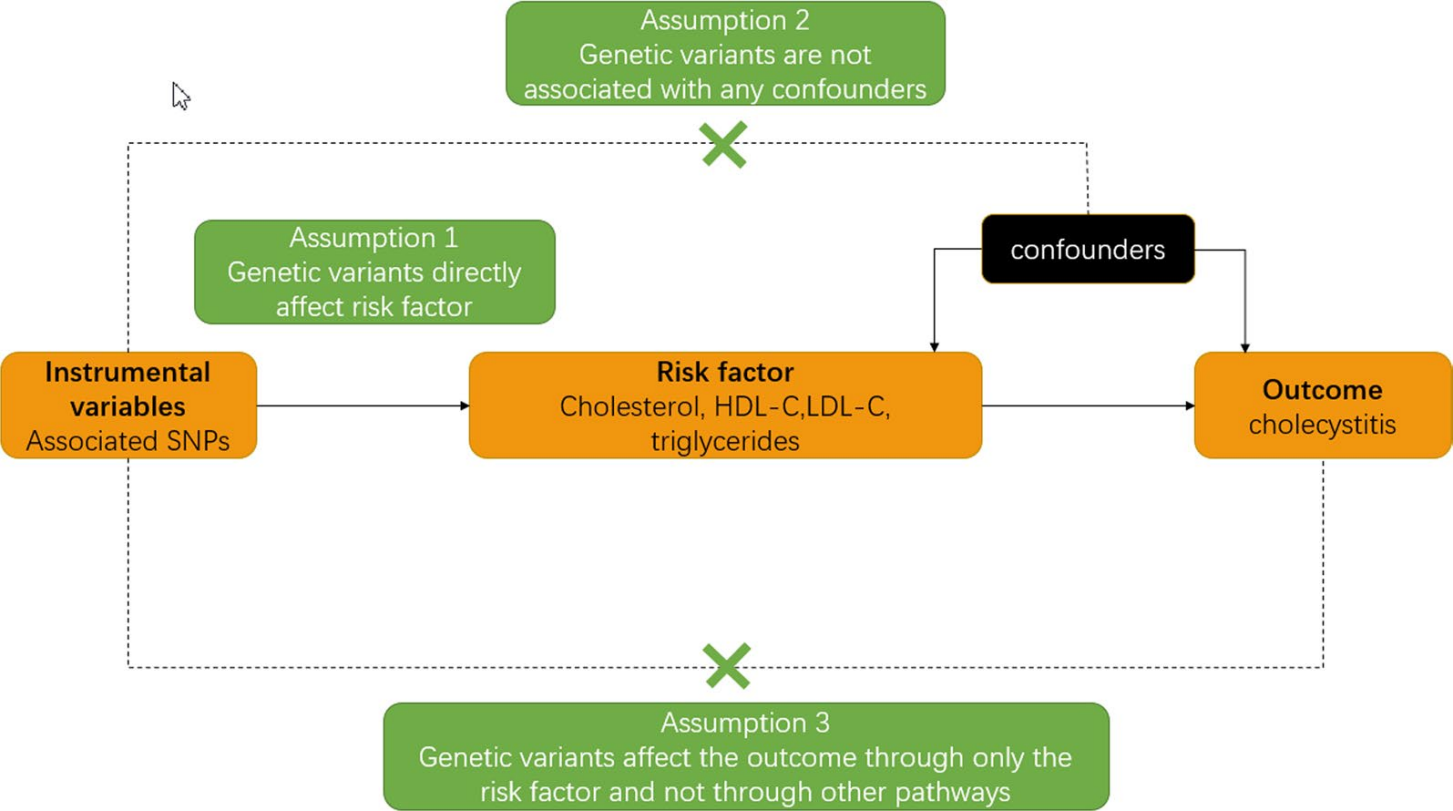
Three principal assumption of MR analysis. *Note*: The dashed lines represent the potential causal relationship between variables that would represent violations of the MR assumptions

Mendelian randomization relies on three assumptions (Fig. 1): (1) the instrument variable (genetic variant) is associated with the risk factor; (2) the instrument variable is not associated with confounders; (3) the instrument variable influences the outcome only by the risk factor. A genetic variant satisfying these assumptions is known as an instrumental variable. SNPs involved in this study could easily fit in assumption 1. We extracted SNPs related to TC, HDL cholesterol, LDL cholesterol and triglycerides as instrumental variables while the SNPs related to cholecystitis were treated as the outcome. With a SNP used as an IV and a biniary outcome, assuming all associations are log-linear, the causal effect of the exposure on the outcome can be estimated as the ratio of the change in the outcome per additional variant allele divided by the change in the risk factor per additional variant allele [10]. Analyses were performed using R, version 4.0.3 (http: //www.r-project.org). R package ‘TwoSampleMR’ and ‘MRPRESSO’ were employed in this study. The R code is provided in this Github repository (https://github.com/YangHQ638/MR-research).

### Statistical analysis

Many specific methods of MR analysis are available. We adopted the following three methods: inverse-variance weighted (IVW), MR-Egger, and weighted median. IVW is the main method adopted in both MR analysis and multivariable MR analysis. IVW estimation is the inverse variance weighted combination of ratio estimates [11] where the random effect model was adopted if there was heterogeneity. Wald ratio is used to obtain a ratio from each SNP. MR-Egger can test and estimate the causal effect. It can also test the directional pleiotropy, making it preferred in the case of pleiotropy. The estimation of MR-Egger is close to that of IVW when the intercept term of MR Egger regression is close to 0 [12]. Compared to IVW (fixed-effect model), the weighted median method could correctly estimate the causality when some of the instrumental variables are invalid [12]. Thus, it is complementary to MR Egger. We have adopted all methods for all exposures. The statistical power of this study was tested by an online tool (https://cnsgenomics.shinyapps.io/mRnd/). To address the issue of multiple testing, results were considered statistically significant at the 0.05 level after a false discovery rate approach of the significance level. We conducted a Cochran Q test to examine the heterogeneity of these analyses. We also tested the pleiotropy by collecting the intercept values from MR Egger regression. The leave-one-out sensitivity analysis was performed to test the robustness of this study.

### Data visualization

We made a scatter plot for the result of each exposure. Different lines of scatter plots represent the causal estimation of different methods. Results of sensitivity analysis were visualized by a leave-one-out plot. The pooled result of all SNPs was shown in the forest plot. Biased was tested by funnel plot [13].

## Result

### IV Validation

The study identified 111 SNPs associated with serum TC level, 123 SNPs associated with HDL cholesterol level, 78 SNPs associated with LDL cholesterol level, and 71 SNPs associated with triglycerides level. All the SNPs were associated with the trait of interest at a genome-wide significant level (*p* < 5 * 10^−8^). Since the summary statistics were missing in the outcome, 2 SNPs related to TC and 2 SNPs related to LDL cholesterol were removed. None of the SNPs was weak instrumental variable (F statistics < 10). 16 SNPs associated with LDL cholesterol were omitted by leave-one-out analysis. A total of 5 palindromic SNPs were excluded. Due to pleiotropy, we removed 3 SNPs related to TC and 2 SNPs related to LDL in the MR-PRESSO test. Excluded SNPs, numbers of identified SNPs, were showed in Table 2. Specific identified SNPs of each phenotype were provided in the Additional materials [see Additional file 1].

**Table 2:** Excluded SNPs, numbers of identified instrumental SNPs

Phenotype	Various types of SNPs excluded				N SNP
	Miss in outcome	Pleiotropy	Leave-one-out	Palindromic structure	
Cholesterol	2	3	2	2	111
HDL-cholesterol	0	0	0	2	123
LDL-cholesterol	1	0	16	3	81
Triglycerides	0	0	0	1	71

*N SNP* number of identified SNPs

### Main results

**Fig. 2:**
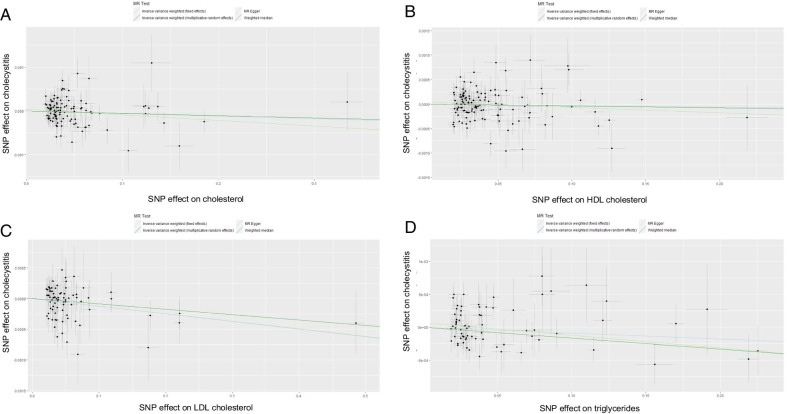
Scatter plots of different lipids. *Note*: Scatter plots showed the causal effect of exposure on cholecystitis. **A** Effect of cholesterol, **B** effect of HDL cholesterol, **C** effect of LDL cholesterol, **D** effect of triglycerides

The causal estimations of MR analysis were provided in Table 3. The minor causal effect of LDL cholesterol on cholecystitis was demonstrated by IVW (multiplicative random effect model) (OR [95% CI] = 0.9984483 [0.9984499, 0.9984468]; *p* = 0.008). There was an evidence of heterogeneity (Q = 116.489, *p* value = 0.004). No evidence was found about pleiotropy (egger intercept =  − 2.1 × 10^−5^, *p* value = 0.659). Although it was not significant at the level of *p* = 0.05, a minor protective effect was observed in TC (OR [95% CI] = 0.9994228 [0.9994222, 0.9994233]; *p* = 0.296), triglycerides (OR [95% CI] = 0.9990893 [0.9990882, 0.9990903]; *p* = 0.238) and HDL cholesterol (OR [95% CI] = 0.9997020 [0.9997017, 0.9997023]; *p* = 0.565). Heterogeneity was detected in HDL cholesterol (Q = 188.737, *p* value = 0.000) and triglycerides (Q = 96.010, *p* value = 0.021) while it was not detected in TC (Q = 135.009, *p* value = 0.053). The MR-Egger intercept suggested there was no evidence of pleiotropy in TC (egger intercept = 4.06 × 10^−5^, *p* value = 0.335), HDL cholesterol (egger intercept = 4.46 × 10^−5^, *p* value = 0.319), and triglycerides (egger intercept = 6.55 × 10^−5^, *p* value = 0.168). The statistical power of these four exposures was relatively low (TC: 0.08, HDL cholesterol: 0.10, LDL cholesterol: 0.08, triglycerides: 0.08). A Scatter plot of MR results was shown in Fig. 2. Forest plots of each SNP were provided in the Additional materials [see Additional file 2].

**Table 3:** The effect estimates, test of heterogeneity and test of pleiotropy of exposure on cholecystitis

	MR methodology	Effect estimates on cholecystitis				Test of heterogeneity		Test of pleiotropy	*p* value
		OR	Lower	Upper	*p* value	Cochrane Q test	*p* value	MR-Egger intercept	
Total cholesterol	Inverse variance weighted (multiplicative random effects)	0.9994228	0.9994222	0.9994233	0.2960	135.009	0.053		
	MR Egger	0.9987183	0.9987161	0.9987205	0.1893	133.856	0.053	0.0000406	0.335
	Inverse variance weighted (fixed effects)	0.9994228	0.9994223	0.9994232	0.2347	135.009	0.053		
	Weighted median	0.9994726	0.9994719	0.9994733	0.5640				
HDL-C	Inverse variance weighted (multiplicative random effects)	0.9997020	0.9997017	0.9997023	0.5650	188.737	0.000		
	MR Egger	0.9988892	0.9988871	0.9988913	0.2510	187.188	0.000	0.0000446	0.319
	Inverse variance weighted (fixed effects)	0.9997020	0.9997018	0.9997023	0.4740	188.737	0.000		
	Weighted median	0.9996111	0.9996106	0.9996117	0.5870				
LDL-C	Inverse variance weighted (multiplicative random effects)	0.9984483	0.9984499	0.9984468	0.0080	116.489188	0.004851838		
	MR Egger	0.9987160	0.9987180	0.9987140	0.1893	116.200524	0.004109598	− 0.000021	0.658974602
	Inverse variance weighted (fixed effects)	0.9984483	0.9984496	0.9984471	0.0008	116.489188	0.004851838		
	Weighted median	0.9991214	0.9991225	0.9991203	0.3380				
Triglycerides	Inverse variance weighted (multiplicative random effects)	0.9990893	0.9990882	0.9990903	0.2380	96.010	0.021		
	MR Egger	0.9980924	0.9980889	0.9980958	0.1680	93.378	0.027	0.0000655	0.168
	Inverse variance weighted (fixed effects)	0.9990893	0.9990884	0.9990902	0.1360	96.010	0.021		
	Weighted median	0.9983449	0.9983424	0.9983474	0.1360				

*TC* total cholesterol, *HDL-C* HDL cholesterol, *LDL-C* LDL cholesterol, *TG* triglycerides, *OR* odds ratio, *upper* upper bounds of 95% confidence interval, *lower* lower bounds of 95% confidence interval. *p* value was adjusted by false discovery rate approach

### Sensitivity analysis

The funnel plots showed the extent of heterogeneity among the individual Wald ratio estimates. The forest plots displayed the effect estimates of each SNP. The leave-one-out plots showed each SNP was robust in MR analysis. The funnel plots, forest plots and leave-one-out plots were provided in the Additional materials [see Additional file 2].

## Discussion

We conducted univariable MR analyses to investigate the causal effect of serum lipids on cholecystitis. The current study found that LDL cholesterol was protective factors against cholecystitis but such protective effect may be subtle because odds ratios were close to 1. The results of HDL cholesterolTC and triglycerides were not statistically significant but all of them showed a protective tendency. The causal association of serum lipids with cholecystitis remains possible, but less certain. Statistical power was relatively low, but nevertheless, the protective effect of LDL cholesterol could still be demonstrated in current study. LDL cholesterol and triglycerides showed a similar trend as a protective factor of cholecystitis, which was partially consistent with some of the previous findings. In univariate analyses conducted by Mohr et al. [3], they found an inverse relationship between HDL cholesterol, LDL cholesterol, and cholecystitis but they also found LDL cholesterol and triglycerides were positively related while HDL cholesterol was not related to cholecystitis in multiple regression. A case–control study conducted by Wang et al. [4] reported that LDL cholesterol was reversely related while HDL cholesterol was positively related. Wang et al. also found a low level of TC increased the risk of gallstone disease [4]. Gallstones can directly induce cholecystitis. Several reports have shown that cholesterol played an important role in the process of gallstone formation [3, 14, 15]. Jiang et al. also reported that patients with gallstone disease had lower serum TC [16]. However, Another case–control study failed to find an association between HDL cholesterol and gallbladder disease [17]. The reason why there are different opinions is that the protective effect of specific serum lipid is really small (but they do exist which is demonstrated in this research). Even some confounding factors are enough to affect the result.

A possible explanation for the protective effect might be the differences in cholesterol transport and secretion. Clinical studies have demonstrated an inverse relationship between serum HDL cholesterol level and bile cholesterol saturation [18]. Lithogenic bile with supersaturated cholesterol is the initial event of the pathogenesis of gallstones and chronic cholecystitis [19, 20]. Supersaturated concentrations of biliary cholesterol cause abnormalities in the mucosa and muscle layers of the gallbladder [19]. They are the results of increase transport of cholesterol across the gallbladder mucosa due to the high concentration in gallbladder [20], which is the result of increasing cholesterol transport from serum to gallbladder. This phenomenon may cause the decrease of serum cholesterol and increase of supersaturated cholesterol biliary simultaneously. Analysis of inbred mice also showed a similar result [21]. Some biochemical analyses have found many molecules related to the transport of cholesterol. SR-BI (scavenger receptor class B type I) is an important determinant of plasma-to-bile transport of HDL cholesterol. Mice with overexpression of SR-BI enhanced biliary cholesterol levels while a decrease of serum cholesterol level was observed in SR-BI deficiency mice [22–25]. SR-BI might upregulate in cholecystitis patients which needs further research to verify. Juvonen et al. reported TaqBI CEPT gene polymorphism, which is correlated with lower serum TC, was associated with cholesterol gallstone disease [14]. It has been demonstrated that gallstone patients secret more cholesterol than normal people [19, 26]. Gallstones could directly lead to cholecystitis. The absolute decrease in serum TC might be a crucial factor in determining cholesterol saturation in bile [27]. However, a long-term high cholesterol diet might cause an increase in serum TC and indirectly increased the secretion of cholesterol [28]. Nevertheless, the way of instrumental variable affect exposure was not the same as the way of diet. So, if we could exclude people with high cholesterol diet, then the result would be clearer. This might be the reason that odds ratio of serum lipids was near to 1. Along with instrumental variables, confounders and environmental factors may co-influence the final result. It is important to conduct a stratified analysis for future research.

Another explanation for our result might be that some of these lipids accelerate gallbladder emptying. Hopman et al. [29] have demonstrated that long-chain triglycerides increased the concentration of plasma cholecystokinin (CCK), which could enhance gallbladder motility while medium-chain triglycerides were not. This might explain that the protective effect of triglycerides was very small. Our results have demonstrated that triglyceride was a minor protective factor. There might be some unknown interactions between different types of cholesterol. Further analyses need to find out the effect of different types of cholesterol and the effect of triglycerides on the gallbladder.

There were still a few drawbacks to our study. Firstly, canalization might have an impact on our result. Due to the whole life-span exposure to low levels of plasma lipids, the body might emerge a negative feedback mechanism to compensate. Secondly, because the instrument variable only could slightly affect the exposure, our result was based on extrapolation. Thirdly, our study was based on a mixed population. Because of different races, cultures, diets, and climate, a study based on a specific population might more persuasive. Moreover, recent epidemiological studies reported women are at a higher risk than men [30], while others believed not [4]. Due to limitations of the Mendelian randomization study, we didn’t investigate the differences between genders. Further studies should be based on a stratified population to overcome this difficulty. Finally, limited by the data source, the statistical power of this research was quite low, hence typeIIerror might occurs in this research. However, it didn’t affect the conclusion that LDL cholesterol was a protective factor against cholecystitis.

Furthermore, previous studies have investigated or stated hypotheses a few underlying mechanisms of lipids affecting cholecystitis. Measuring serum lipids is much easier than measuring cholesterol in the gallbladder in clinical practice. Future studies should not only focus on the relationship between cholesterol saturation and cholecystitis but also investigate the relationship between serum lipids and cholecystitis.

In conclusion, the minor protective effect of LDL cholesterol has been demonstrated in this MR analysis. The protective effect of HDL cholesterol, triglycerides and TC on cholecystitis were also found but not statistically significant in this study. However, the protective effect demonstrated in this study is negligible. The protective effect of serum lipids on cholecystitis, though possible, remain less certain.

## Supplementary Information

**Additional file 1.** Identified SNPS. Identified SNPS for each exposure (TC, HDL cholesterol, LDL cholesterol, triglycerides).

**Additional file 2.** None. Funnel plots, forest plots and leave-one-out plots for each exposure (TC, HDL cholesterol, LDL cholesterol, triglycerides).

## Data Availability

The data used to support the findings of this study are included within the article.

## Abbreviations

LDL: Low-density lipoprotein
HDL: High-density lipoprotein
TC: Total cholesterol
MR: Mendelian randomization
SNP: Single nucleotide polymorphisms
GWAS: Genome-wide association studies
HWE: Hardy–Weinberg equilibrium
