# Supplementary material for: Mendelian randomization rules out the causal relationship between serum lipids and cholecystitis
Source: BMC Med Genomics. 2021 Sep 17;14:224. doi: 10.1186/s12920-021-01082-y (PMC8447629; doi:10.1186/s12920-021-01082-y)
Supplement: Supplementary file 2 — Additional file 2. None. Funnel plots, forest plots and leave-one-out plots for each exposure (TC, HDL cholesterol, LDL cholesterol, triglycerides). [file 12920_2021_1082_MOESM2_ESM.pdf]

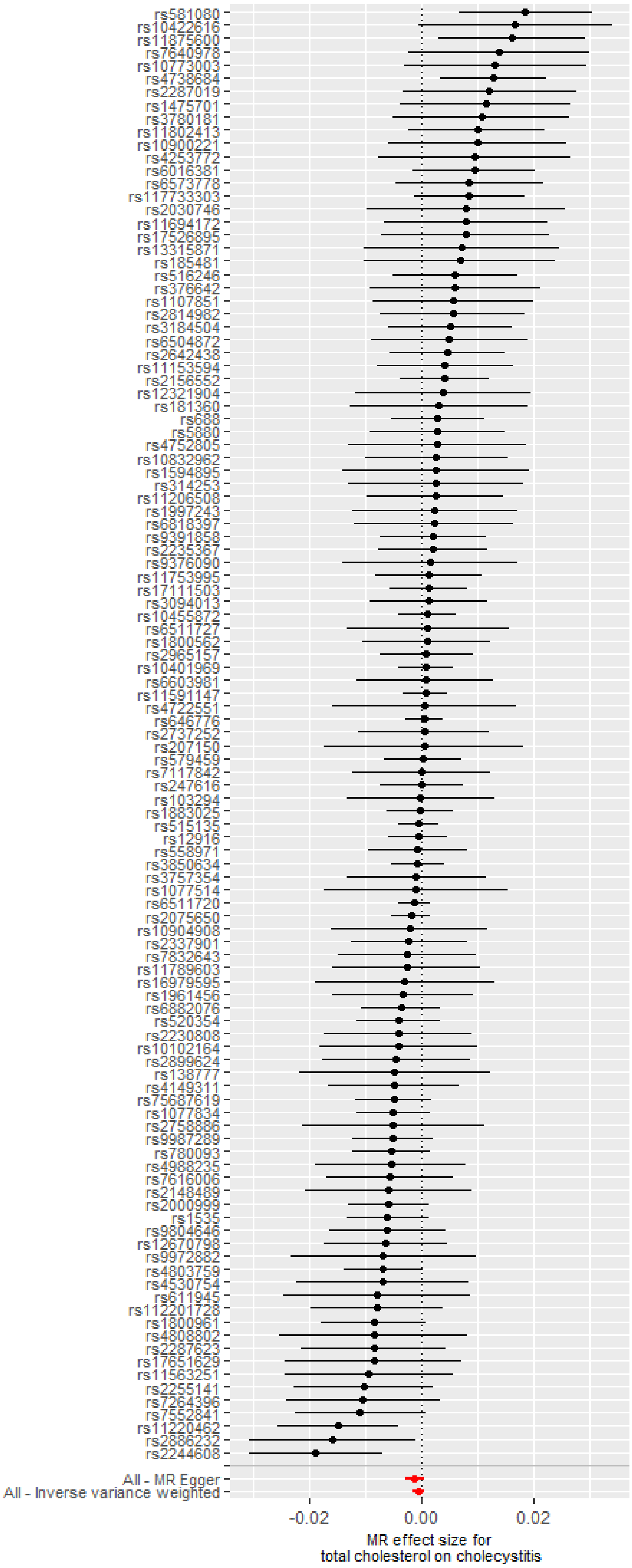
rs581080
rs10422616
rs11875600
rs7640978
rs10773003
rs4738684
rs2287019
rs1475701
rs3780181
rs11802413
rs10900221
rs4253772
rs6016381
rs6573778
rs117733303
rs2030746
rs11694172
rs17526895
rs13315871
rs185481
rs516246
rs376642
rs1107851
rs2814982
rs3184504
rs6504872
rs2642438
rs11153594
rs2156552
rs12321904
rs181360
rs688
rs5880
rs4752805
rs10832962
rs1594895
rs314253
rs11206508
rs1997243
rs6818397
rs9391858
rs2235367
rs9376090
rs11753995
rs17111503
rs3094013
rs10455872
rs6511727
rs1800562
rs2965157
rs10401969
rs6603981
rs11591147
rs4722551
rs646776
rs2737252
rs207150
rs579459
rs7117842
rs247616
rs103294
rs1883025
rs515135
rs12916
rs558971
rs3850634
rs3757354
rs1077514
rs6511720
rs2075650
rs10904908
rs2337901
rs7832643
rs11789603
rs16979595
rs1961456
rs6882076
rs520354
rs2230808
rs10102164
rs2899624
rs138777
rs4149311
rs75687619
rs1077834
rs2758886
rs9987289
rs780093
rs4988235
rs7616006
rs2148489
rs2000999
rs1535
rs9804646
rs12670798
rs9972882
rs4803759
rs4530754
rs611945
rs112201728
rs1800961
rs4808802
rs2287623
rs17651629
rs11563251
rs2255141
rs7264396
rs7552841
rs11220462
rs2886232
rs2244608
All - MR Egger
All - Inverse variance weighted
-0.02
0.00
0.02
MR effect size for
total cholesterol on cholecystitis

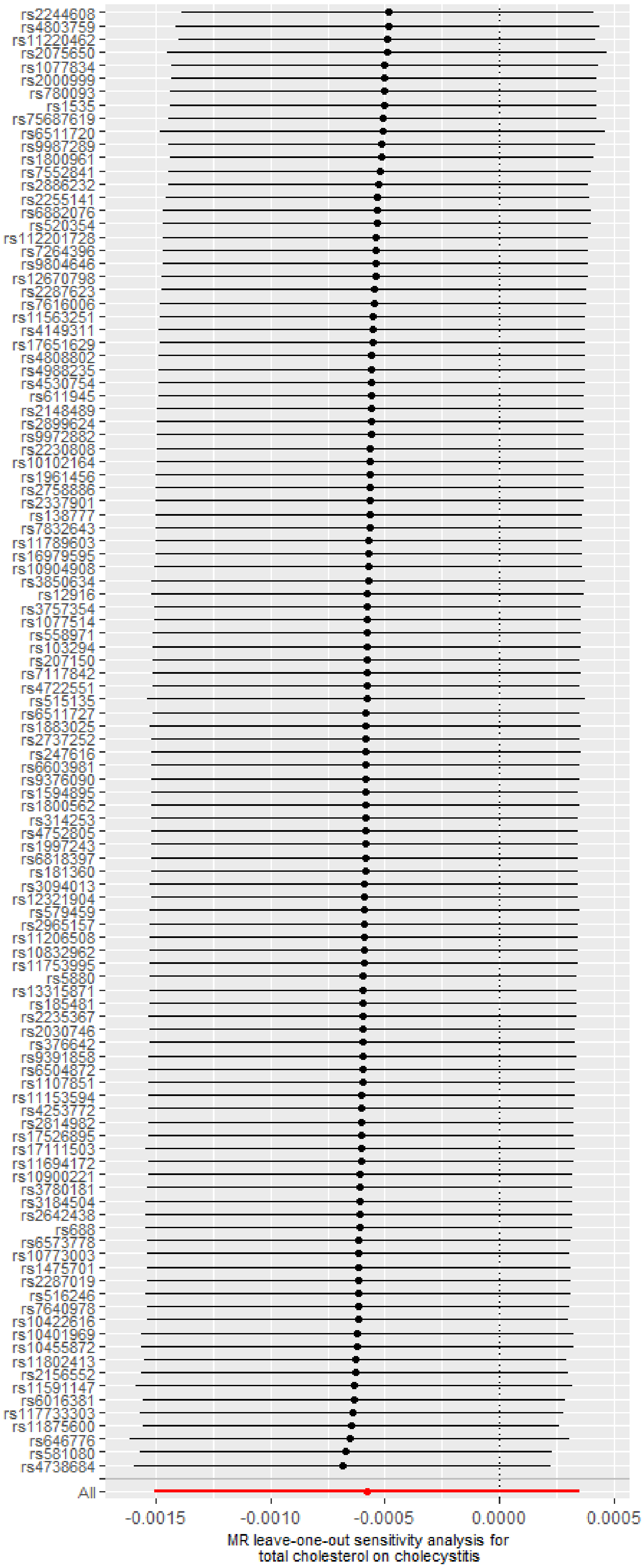
rs2244608
rs4803759
rs11220462
rs2075650
rs1077834
rs2000999
rs780093
rs1535
rs75687619
rs6511720
rs9987289
rs1800961
rs7552841
rs2888232
rs2255141
rs6882076
rs520354
rs112201728
rs7264396
rs9804646
rs12670798
rs2287623
rs7616006
rs11563251
rs4149311
rs17651629
rs4808802
rs4988235
rs4530754
rs611945
rs2148489
rs2899624
rs9972882
rs2230808
rs10102164
rs1961456
rs2758886
rs2337901
rs138777
rs7832643
rs11789603
rs16979595
rs10904908
rs3850634
rs12916
rs3757354
rs1077514
rs558971
rs103294
rs207150
rs7117842
rs4722551
rs515135
rs6511727
rs1883025
rs2737252
rs247616
rs6603981
rs9376090
rs1594895
rs1800562
rs314253
rs4752805
rs1997243
rs6818397
rs181360
rs3094013
rs12321904
rs579459
rs2965157
rs11206508
rs10832962
rs11753995
rs5880
rs13315871
rs185481
rs2235367
rs2030746
rs376642
rs9391858
rs6504872
rs1107851
rs11153594
rs4253772
rs2814982
rs17526895
rs17111503
rs11694172
rs10900221
rs3780181
rs3184504
rs2642438
rs688
rs6573778
rs10773003
rs1475701
rs2287019
rs516246
rs7640978
rs10422616
rs10401969
rs10455872
rs11802413
rs2156552
rs11591147
rs6016381
rs117733303
rs11875600
rs646776
rs581080
rs4738684
All
-0.0015
-0.0010
-0.0005
0.0000
0.0005
MR leave-one-out sensitivity analysis for
total cholesterol on cholecystitis

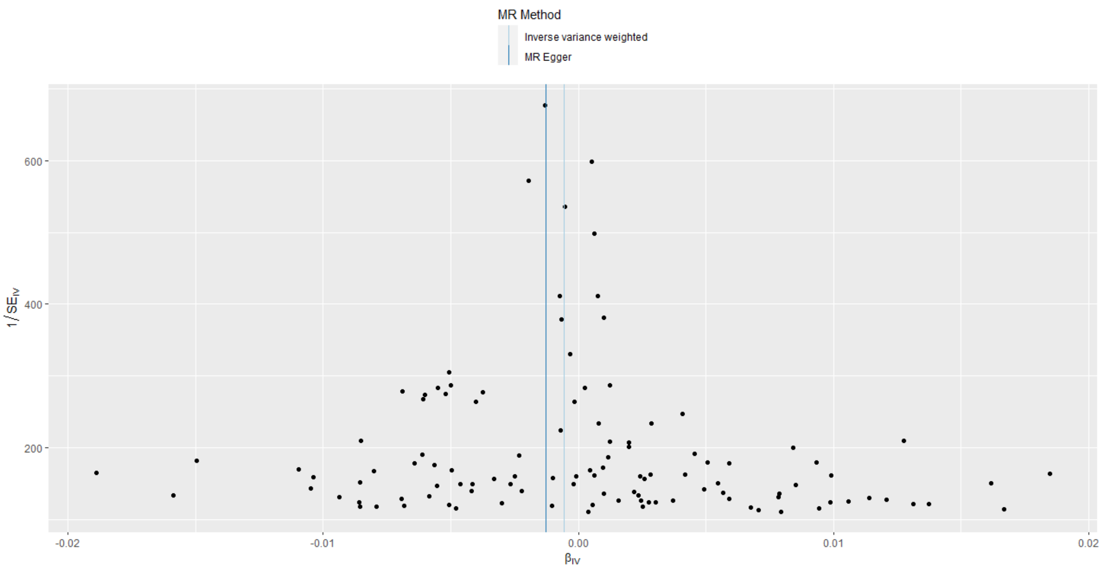

Funnel plot for total cholesterol

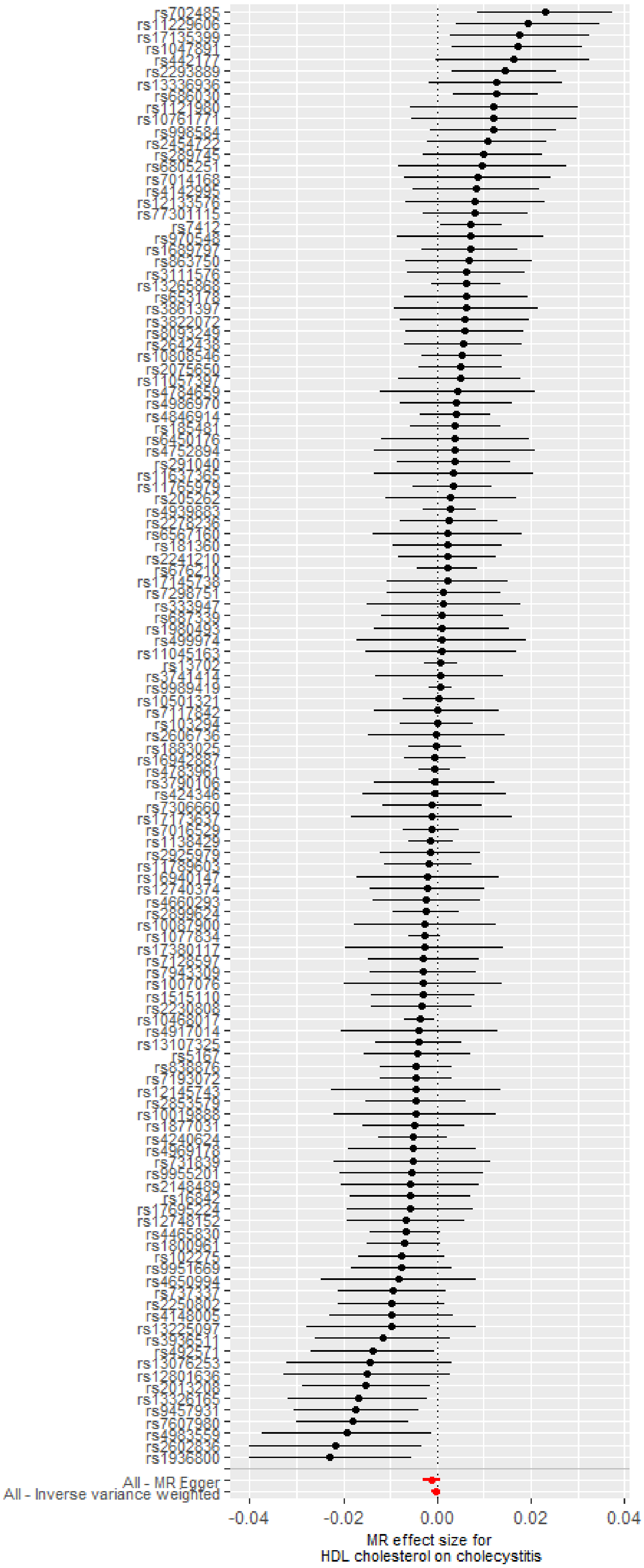
All - MR Egger
All - Inverse variance weighted
-0.04
-0.02
0.00
0.02
0.04
MR effect size for
HDL cholesterol on cholecystitis

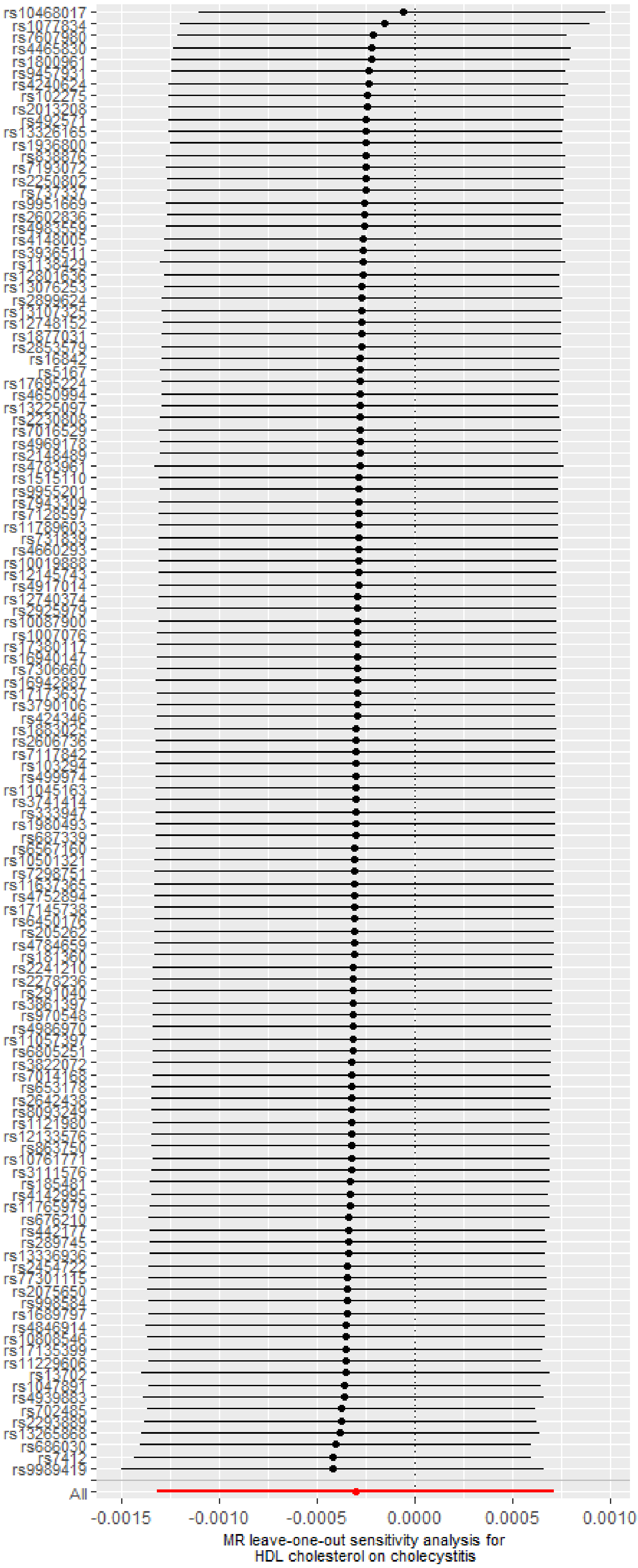
All
-0.0015
-0.0010
-0.0005
0.0000
0.0005
0.0010
MR leave-one-out sensitivity analysis for
HDL cholesterol on cholecystitis

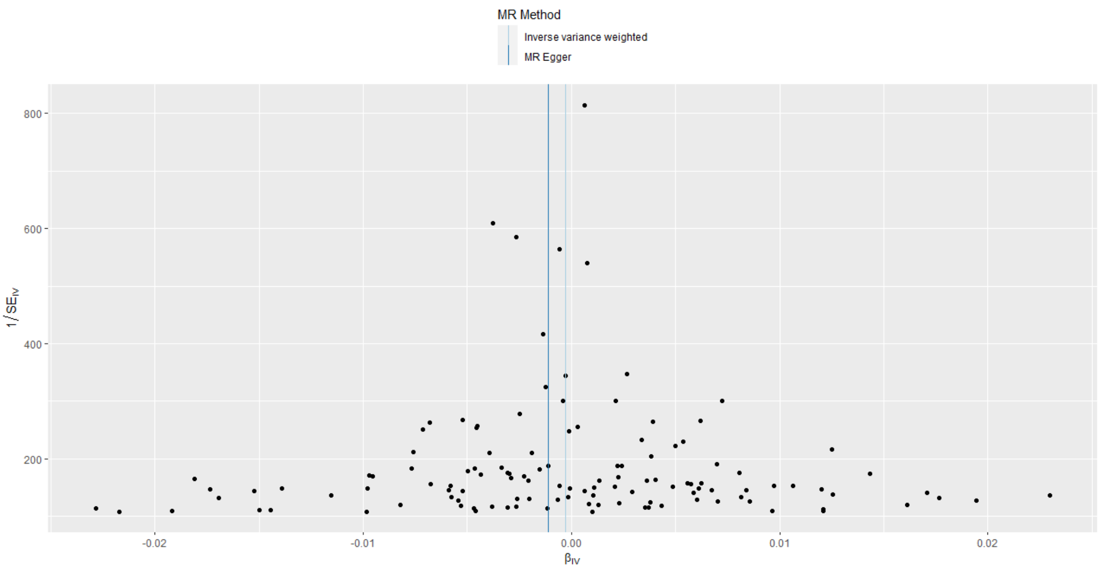

Funnel plot for HDL cholesterol

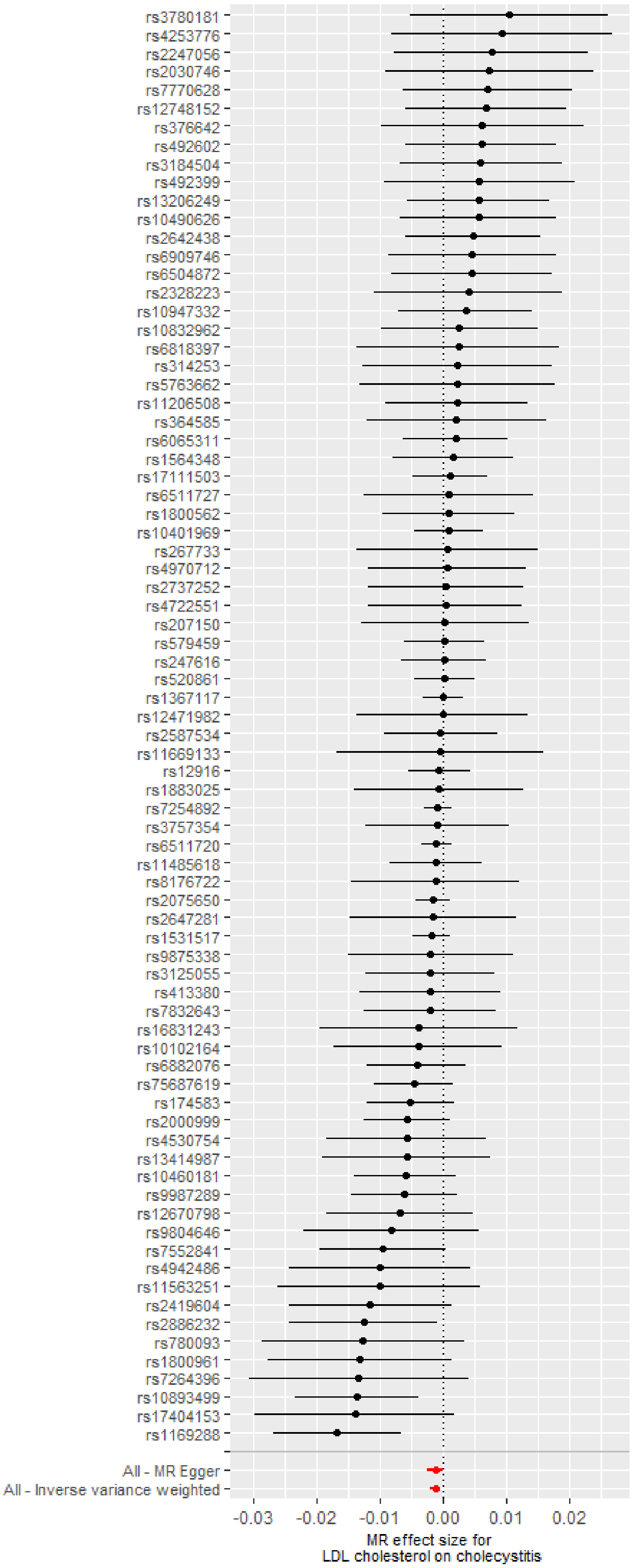
rs3780181
rs4253776
rs2247056
rs2030746
rs7770628
rs12748152
rs376642
rs492602
rs3184504
rs492399
rs13206249
rs10490626
rs2642438
rs6909746
rs6504872
rs2328223
rs10947332
rs10832962
rs6818397
rs314253
rs5763662
rs11206508
rs364585
rs6065311
rs1564348
rs17111503
rs6511727
rs1800562
rs10401969
rs267733
rs4970712
rs2737252
rs4722551
rs207150
rs579459
rs247616
rs520861
rs1367117
rs12471982
rs2587534
rs11669133
rs12916
rs1883025
rs7254892
rs3757354
rs6511720
rs11485618
rs8176722
rs2075650
rs2647281
rs1531517
rs9875338
rs3125055
rs413380
rs7832643
rs16831243
rs10102164
rs6882076
rs75687619
rs174583
rs2000999
rs4530754
rs13414987
rs10460181
rs9987289
rs12670798
rs9804646
rs7552841
rs4942486
rs11563251
rs2419604
rs2886232
rs780093
rs1800961
rs7264396
rs10893499
rs17404153
rs1169288
All - MR Egger
All - Inverse variance weighted
-0.03
-0.02
-0.01
0.00
0.01
0.02
MR effect size for
LDL cholesterol on cholecystitis

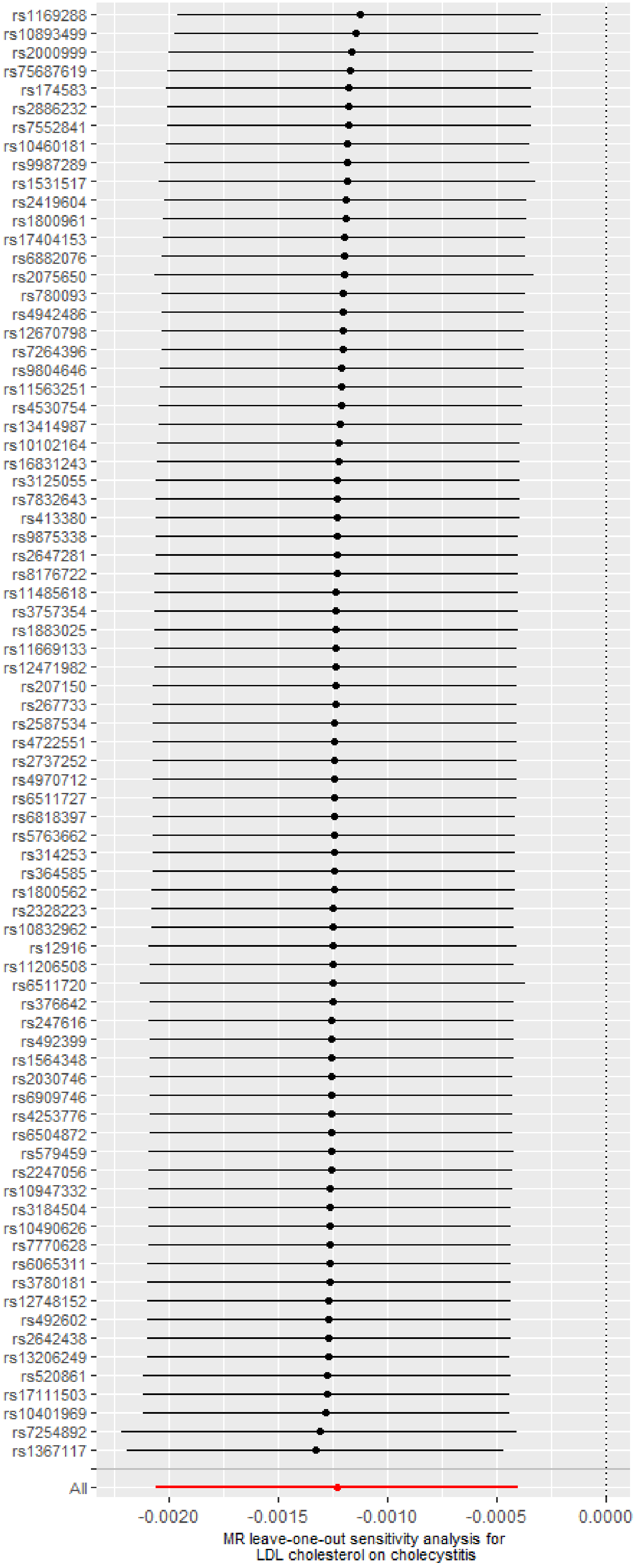
rs1169288
rs10893499
rs2000999
rs75687619
rs174583
rs2886232
rs7552841
rs10460181
rs9987289
rs1531517
rs2419604
rs1800961
rs17404153
rs6882076
rs2075650
rs780093
rs4942486
rs12670798
rs7264396
rs9804646
rs11563251
rs4530754
rs13414987
rs10102164
rs16831243
rs3125055
rs7832643
rs413380
rs9875338
rs2647281
rs8176722
rs11485618
rs3757354
rs1883025
rs11669133
rs12471982
rs207150
rs267733
rs2587534
rs4722551
rs2737252
rs4970712
rs6511727
rs6818397
rs5763662
rs314253
rs364585
rs1800562
rs2328223
rs10832962
rs12916
rs11206508
rs6511720
rs376642
rs247616
rs492399
rs1564348
rs2030746
rs6909746
rs4253776
rs6504872
rs579459
rs2247056
rs10947332
rs3184504
rs10490626
rs7770628
rs6065311
rs3780181
rs12748152
rs492602
rs2642438
rs13206249
rs520861
rs17111503
rs10401969
rs7254892
rs1367117
All
-0.0020
-0.0015
-0.0010
-0.0005
0.0000
MR leave-one-out sensitivity analysis for
LDL cholesterol on cholecystitis

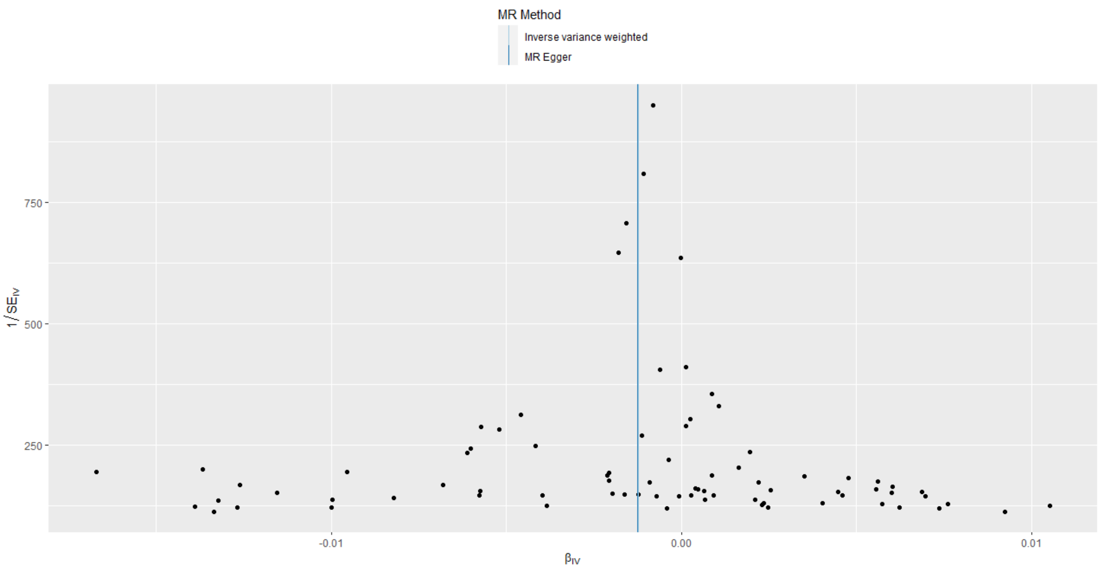

Funnel plot for LDL cholesterol

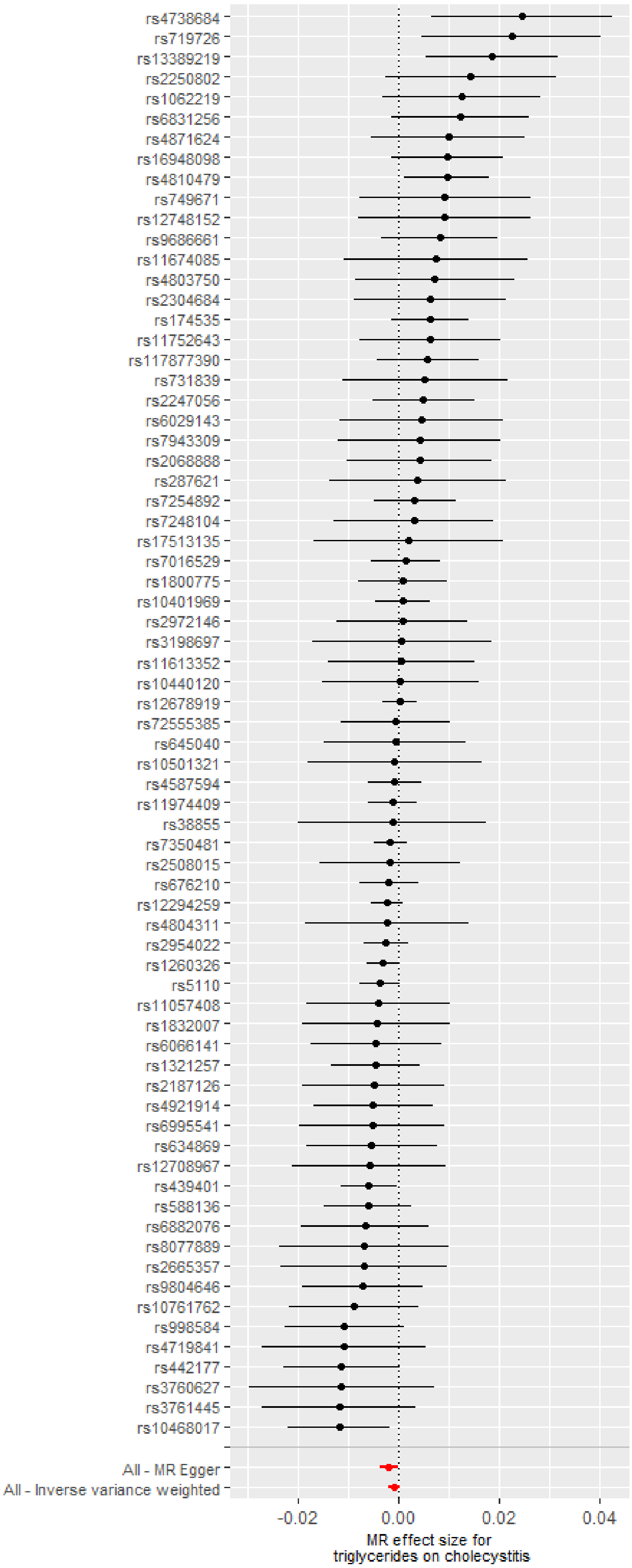
rs4738684
rs719726
rs13389219
rs2250802
rs1062219
rs6831256
rs4871624
rs16948098
rs4810479
rs749671
rs12748152
rs9686661
rs11674085
rs4803750
rs2304684
rs174535
rs11752643
rs117877390
rs731839
rs2247056
rs6029143
rs7943309
rs2068888
rs287621
rs7254892
rs7248104
rs17513135
rs7016529
rs1800775
rs10401969
rs2972146
rs3198697
rs11613352
rs10440120
rs12678919
rs72555385
rs645040
rs10501321
rs4587594
rs11974409
rs38855
rs7350481
rs2508015
rs676210
rs12294259
rs4804311
rs2954022
rs1260326
rs5110
rs11057408
rs1832007
rs6066141
rs1321257
rs2187126
rs4921914
rs6995541
rs634869
rs12708967
rs439401
rs588136
rs6882076
rs8077889
rs2665357
rs9804646
rs10761762
rs998584
rs4719841
rs442177
rs3760627
rs3761445
rs10468017
All - MR Egger
All - Inverse variance weighted
-0.02
0.00
0.02
0.04
MR effect size for
triglycerides on cholecystitis

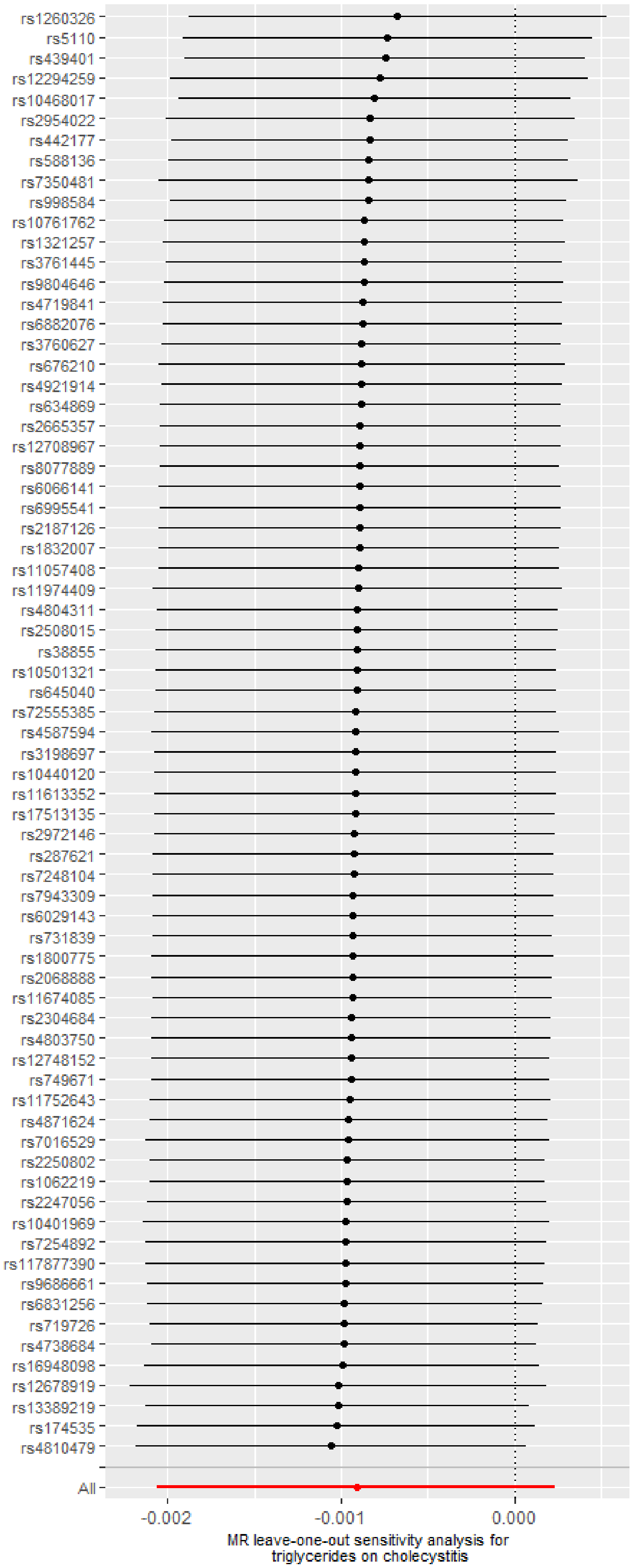

rs1260326
rs5110
rs439401
rs12294259
rs10468017
rs2954022
rs442177
rs588136
rs7350481
rs998584
rs10761762
rs1321257
rs3761445
rs9804646
rs4719841
rs6882076
rs3760627
rs676210
rs4921914
rs634869
rs2665357
rs12708967
rs8077889
rs6066141
rs6995541
rs2187126
rs1832007
rs11057408
rs11974409
rs4804311
rs2508015
rs38855
rs10501321
rs645040
rs72555385
rs4587594
rs3198697
rs10440120
rs11613352
rs17513135
rs2972146
rs287621
rs7248104
rs7943309
rs6029143
rs731839
rs1800775
rs2068888
rs11674085
rs2304684
rs4803750
rs12748152
rs749671
rs11752643
rs4871624
rs7016529
rs2250802
rs1062219
rs2247056
rs10401969
rs7254892
rs117877390
rs9686661
rs6831256
rs719726
rs4738684
rs16948098
rs12678919
rs13389219
rs174535
rs4810479
All
-0.002
-0.001
0.000
MR leave-one-out sensitivity analysis for
triglycerides on cholecystitis

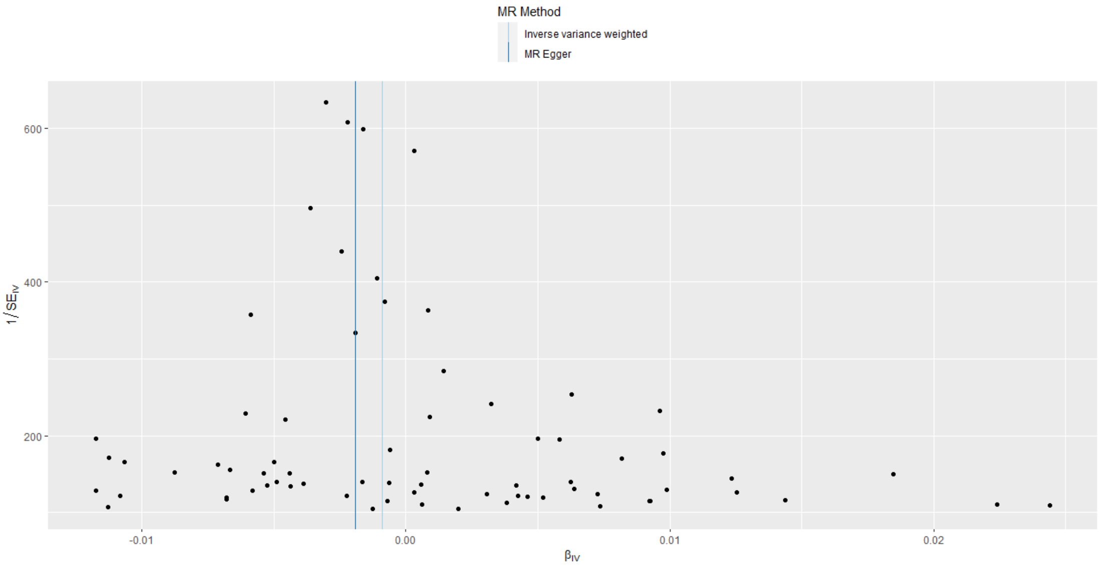

Funnel plot for triglycerides
